# Integrating mental health and disaster preparedness in intervention: a randomized controlled trial with earthquake and flood-affected communities in Haiti

**DOI:** 10.1017/S0033291719000163

**Published:** 2019-02-14

**Authors:** Leah Emily James, Courtney Welton-Mitchell, John Roger Noel, Alexander Scott James

**Affiliations:** 1 Institute of Behavioral Science, Natural Hazards Center, University of Colorado-Boulder, 483 UCB, Boulder, CO 80309-0483, USA; 2 Institute of Behavioral Science, Natural Hazards Center, University of Colorado-Boulder, 483 UCB, Boulder, CO 80309-0483, USA; 3 Soulaje Lespri Moun, Port-au-Prince, Haiti; 4 Statistician/Independent Consultant, New York, NY, USA

**Keywords:** Disaster, flood, earthquake, Haiti, intervention, mental health, preparedness

## Abstract

**Background:**

Given the frequency of natural hazards in Haiti, disaster risk reduction is crucial. However, evidence suggests that many people exposed to prior disasters do not engage in disaster preparedness, even when they receive training and have adequate resources. This may be partially explained by a link between mental health symptoms and preparedness; however, these components are typically not integrated in intervention.

**Methods:**

The current study assesses effectiveness of an integrated mental health and disaster preparedness intervention. This group-based model was tested in three earthquake-exposed and flood-prone communities (*N* = 480), across three time points, using a randomized controlled trial design. The 3-day community-based intervention was culturally-adapted, facilitated by trained Haitian lay mental health workers, and focused on enhancing disaster preparedness, reducing mental health symptoms, and fostering community cohesion.

**Results:**

Consistent with hypotheses, the intervention increased disaster preparedness, reduced symptoms associated with depression, post-traumatic stress disorder, anxiety, and functional impairment, and increased peer-based help-giving and help-seeking. Mediation models indicated support for the underlying theoretical model, such that the effect of the intervention on preparedness was mediated by mental health, and that effects on mental health were likewise mediated by preparedness.

**Conclusions:**

The community-based mental health-integrated disaster preparedness intervention is effective in improving mental health and preparedness among community members in Haiti vulnerable to natural hazards. This brief intervention has the potential to be scaled up for use with other communities vulnerable to earthquakes, seasonal flooding, and other natural hazards.

## Introduction

In recent years, the UN's head of disaster planning has warned that a global failure to plan for future natural disasters will have ‘inconceivably bad’ consequences, especially as climate change leads to increase in chronically-occurring natural disasters and the humanitarian crises that often result (Jones, 2016). The UN and others have called for increased attention to preparedness efforts, which receive just 0.4% of the global aid budget (in 2014). Others have warned that extreme weather events fueled by climate change will have an increasingly disastrous effect on mental health, constituting a public health crisis requiring immediate intervention (e.g. Shukla, 2013). Given this, it is essential to understand factors contributing both to recovery from disaster impact and to preparedness for future disasters, and to develop and test interventions that take these factors into account.

In chronically disaster-exposed contexts, engagement in individual and community-level disaster preparedness is critical. Even in resource-poor settings, low-cost options exist, such as planning evacuation routes, identifying community vulnerabilities and resources, practicing safe storage of documents, creating preparedness kits comprised of basic supplies, and teaching children about disaster response strategies. Yet, despite substantial efforts to train disaster-prone communities in disaster risk reduction strategies, growing evidence suggests that many people do not engage in preparedness activities, even when they possess sufficient resources, receive related education, and/or have a history of disaster exposure (Miller, 2012; Donahue *et al*., 2014; Petkova *et al*., 2016). Moreover, some research suggests that rather than increasing motivation to prepare, in certain cases a prior history of disaster exposure may actually make one less likely to prepare, and that reasons for this are sometimes psychological in nature (Morrissey and Reser, 2003; Lin *et al*., 2008; Paton *et al*., 2009; Solberg *et al*., 2010; Mishra and Suar, 2012; James, 2013).

Mental health symptoms, including those associated with prior disaster exposure, may influence preparedness for future disasters. Disasters can have severe mental health consequences, stemming from exposure to potentially traumatic events and to ongoing stressors exacerbated by the disaster, especially for those with limited resources to support recovery (Norris *et al*., 2002; WHO and PAHO, 2010). Reviews and meta-analyses place prevalence of post-traumatic stress disorder (PTSD) at 30–40% among direct disaster survivors (Goldmann and Galea, 2014), while depression rates range from 6% to 54%, depending on survivor and disaster characteristics (Tang *et al*., 2014). Although less studied, generalized anxiety disorder symptoms are also reported (McFarlane *et al*., 2009).

Depression, PTSD, and anxiety may influence preparedness through a variety of mechanisms. The symptom profile of depression includes hopelessness which may reduce motivation to prepare by implying that one is incapable of creating desired outcomes, or that the worst will occur regardless of one's efforts to the contrary (Lin *et al*., 2008; Bodas *et al*., 2017). Those who have experienced severe distress associated with prior disasters may avoid any engagement with disaster-related content, including thoughts or behaviors associated with future disasters, thus impeding preparedness efforts – a reaction consistent with avoidance symptoms typically associated with PTSD (James, 2013). Anxiety has been linked to reduced preparedness for flood and heatwaves in an Indian population (Mishra and Suar, 2012) and reduced preparedness for conflict in an Israeli population (Bodas *et al*., 2017).

Across diagnostic categories, functional impairment related to mental health symptoms may also play an important role. In a meta-analysis of disaster survivors in 80 countries, nearly one-quarter of those reporting mental health symptoms also experienced substantial functional impairment, limiting their ability to perform daily activities (Norris *et al*., 2002). It follows that such impairment may not only impact one's ability to recover from disaster, but also to engage in ongoing disaster preparedness (e.g. Eisenman *et al*., 2009).

Given this, it is plausible that disaster preparedness interventions may be more effective when they also address disaster-related mental health symptoms (Acharya *et al*., 2006; James, 2013). Specifically, interventions can teach coping skills to improve functioning, reduce hopelessness, and enhance participants’ ability to engage with, rather than avoid, potentially anxiety-inducing disaster preparedness content (during the intervention itself and when implementing preparedness at home).

Just as improved mental health may encourage increased preparedness, preparedness behaviors may in turn positively impact mental health. To the extent that preparedness reduces negative impact of future disasters, it also protects against associated mental health consequences of impending disasters. Moreover, concerns about future disasters can be a significant source of ongoing distress for survivors of chronic disasters; feeling adequately prepared may increase efficacy and perceived safety, and so decrease feelings of anxiety and depression (e.g. Galappatti and Richardson, 2016; Takahashi and Kitamura, 2016). Although little research has directly investigated this link, it follows that mental health-focused interventions for disaster vulnerable populations may benefit from inclusion of disaster risk reduction content.

Community cohesion, and associated social support, is likely to also play a role in recovery and disaster preparedness, and in mental health and well-being (Hikichi *et al*., 2016; Welton-Mitchell *et al*., 2016). There is a rich literature on the link between social support and mental well-being across the lifespan, including in times of adversity (see Siedlecki *et al*., 2014 for a review). The strength of a community's social networks and the extent to which it operates cohesively can affect the well-being of individual community members, as well as the ability of the entire community to address natural hazards through coordinated efforts (Nakagawa and Shaw, 2004; Rodriguez *et al*., 2006; Afifi *et al*., 2012; Adeola and Picou, 2014; Toya and Skidmore, 2014). However, disasters may disrupt social networks, interfering with support and undermining trust (Oyama *et al*., 2012; Albrecht, 2018), so increasing vulnerability when future disasters occur. Group interventions which emphasize exchange of peer-support and encourage peer-based help-seeking and help-giving may enhance social cohesion and social support (Layne *et al*., 2001; Hogan *et al*., 2002), so benefiting both preparedness and mental health outcomes.

### Study site

Port-au-Prince was chosen as the research site due to its extreme vulnerability to disasters, including earthquakes, hurricanes, flooding and landslides (see online Supplementary Appendix 1 for details). Findings from research with survivors of the 2010 earthquake in Haiti indicate high rates of PTSD, depression and other forms of distress, yet access to effective treatments is rare (James *et al*., 2012; Cerdá *et al*., 2013).

### Hypothesized effects

In chronically disaster-prone contexts, it is important to focus on promoting recovery from past disasters and encouraging preparedness for future disasters. We hypothesize that a brief intervention that integrates disaster preparedness content with mental health psychoeducation and coping skills, as well as a framework that encourages social cohesion and peer support, is likely to be especially effective in such settings. To this end, we developed a 3-day *mental health integrated disaster preparedness* group intervention for vulnerable communities in metropolitan Port-au-Prince, Haiti and evaluated it using a randomized controlled trial (RCT) design.

This study tested the following hypothesized intervention effects: (1) increase in engagement in disaster preparedness activities; (2) decrease in depression, PTSD (overall and avoidance sub-cluster), and generalized anxiety^1^^†^, and in mental health-related functional impairment; (3) increase in perceived social cohesion; (4) increase in willingness to provide mental health and disaster-related help to others; and (5) increase in willingness to engage in mental health and disaster preparedness-related help-seeking.

### Underlying theoretical model: hypothesized role of disaster exposure

In addition to investigating intervention outcomes, the current study aims to elucidate support for the underlying theoretical model. Specifically, we investigate the premise that prior disaster exposure can be associated with reduced preparedness activities, particularly among those suffering from mental health symptoms. We hypothesize correlations among prior disaster exposure and disaster preparedness and mental health symptoms at baseline (Time 1), such that greater disaster exposure is associated with (6) reduced preparedness and (7) increased mental health symptoms. Further, we predict (8) mediated effects (at Time 1) such that the relationship between disaster exposure and reduced preparedness is explained by heightened mental health symptoms.

### Hypothesized mechanisms of intervention change

Finally, we investigate potential change pathways for intervention effects. We hypothesize correlations among disaster preparedness, mental health symptoms, and social cohesion at Time 1, such that (9) less severe mental health symptoms are associated with increased preparedness, and (10) social cohesion is positively associated with both mental health and preparedness. Further, we hypothesize mediated intervention effects such that (11) a change in mental health (depression, PTSD, anxiety, and functional impairment) will explain the impact of the intervention on preparedness. As little work has been done in this area, we will also test a reversed model to determine if (12) a change in preparedness will explain the impact of the intervention on mental health. Finally, we predict (13) a change in social cohesion will explain the impact of the intervention on preparedness and on mental health.

## Method

This study was reviewed and approved by the Institutional Review Board at the University of Colorado, Boulder, as well as Enstite Travay Sosyal ak Syens Sosyal (ETS; Institute of Social Work and Social Science) in Port-au-Prince.

### Participants and procedures

Research included 480 randomly selected community members, drawn from three disaster-affected communities (160 participants in each) in metropolitan Port-au-Prince between July 2014 and April 2015. Identification of research communities and participant recruitment procedures are detailed in online Supplementary Appendix 1.

### Design

Research utilized a RCT design. All participants (480 total) completed assessment interviews at baseline (Time 1; July–August 2014) including outcome measures, demographic factors, and indicators of vulnerability (see Measures section below for details). After all completed baseline interviews, half of participants from each community (equal gender representation) were randomly selected to receive the 3-day intervention soon afterward (August 2014). Randomization occurred using a random number generator applied to participant lists. Due to staffing constraints, interviewers were not blind to condition, as team members served as both interviewers and intervention facilitators (though participants were not typically interviewed by the same staff person who facilitated their group's intervention). Members of the research team observed intervention sessions, completing a fidelity checklist to ensure that key components were covered.

Over the next several months, Port-au-Prince experienced a typical hurricane season with moderate associated flooding and other storm-related damage in the research communities. In December 2014 (3–4 months after the first assessment), Time 2 data collection was conducted for all (treatment and control group) participants using the same measures employed at baseline. In March 2015, all participants completed a third (Time 3; follow-up) assessment, again using a similar interview schedule. In August 2015, following the third data collection timepoint, wait-list control group participants were invited to participate in the intervention.

### Intervention

In each of the three communities, four 3-day intervention groups of no more than 20 participants each were facilitated by two trained Haitian lay mental health workers. The *mental health integrated disaster preparedness* intervention utilizes an experiential approach, including facilitated discussion, space for sharing personal experiences and exchange of peer-support, establishing safety and practicing coping skills targeting disaster-related distress, and hands-on training in disaster preparedness and response techniques for use by participants in their own lives and to support other community members. Content was developed in collaboration with Haitian team members, using an approach consistent with best practice guidelines and recommendations from others working in Haiti (see Ferrer-Wreder *et al*., 2012; Guerda *et al*., 2015). The content of the sessions is based on a standardized manual (James *et al*., 2016, publicly available online in English and Haitian Kreyol).

Details are available in online Supplementary Appendix 2 and in other publications (James *et al*., 2018; Welton-Mitchell *et al*., 2018; Welton-Mitchell and James, 2018).

### Measures

Haitian researchers employed by Soulaje Lespri Moun conducted participant interviews in Haitian Kreyol. Data were collected using Qualtrics survey software loaded onto handheld tablets. Structured interviews took 45–60 min. A subset of variables, constituting primary outcomes, is examined in this manuscript. When possible, culturally-adapted and validated measures were used (see Table 1 for brief description of measures and online Supplementary Appendix 3 for detailed measures and reliability statistics).

**Table 1.** Baseline characteristics for full sample and for intervention and control groups

				Analysis
Characteristics	Total sample (*n* = 480)	Intervention (*n* = 240)	Control (*n* = 240)	*p* (intervention *v*. control)
Female gender, *n* (%)	239 (49.8%)	117 (48.8%)	122 (50.8%)	0.72
Mean age, years (s.d.), range	37 (13.6), 18–78	36 (12.7), 18–75	38 (14.3), 18–78	0.09
Currently married, *n* (%)	116 (24.6%)	52 (21.9%)	64 (27.2%)	0.20
Mean number of children (s.d.), range	2.6 (2.5), 0–14	2.4 (2.5), 0–14	2.9 (2.5), 0–13	0.03
Currently employed, *n* (%)	27 (5.7%)	14 (5.9%)	13 (5.5%)	1.00
Currently in school, *n* (%)	167 (35.4%)	80 (33.9%)	87 (36.9%)	0.56
Mean education, years (s.d.), range	7.3 (4.5), 0–20	7.3 (4.7), 0–20	7.2 (4.4), 0–16	0.73
Religion, *n* (%)				0.12
Protestant	186 (40.1%)	86 (37.1%)	100 (43.1%)	
Catholic	148 (31.9%)	69 (29.7%)	79 (34.1%)	
Other Christian	90 (19.2%)	53 (22.8%)	37 (15.5%)	
Mean chronic stressors^a^ (s.d.), range	10.3 (5.8) 0–24	10.1 (6.0) 0–24	10.5 (5.6) 0–24	0.46
Mean disaster exposure scale^b^ (s.d.), range	5.7 (2.2) 0–11	5.9 (2.2) 0–11	5.6 (2.3) 1–11	0.20
Earthquake exposure, *n* (%)	469 (98.1%)	234 (97.5%)	235 (98.7%)	0.50
Flood exposure, *n* (%)	316 (66.2%)	159 (66.5%)	157 (66.0%)	0.92
Significant property damage, *n* (%)	338 (70.7%)	177 (73.8%)	161 (67.7%)	0.16
Displaced, *n* (%)	274 (57.7%)	137 (57.8%)	137 (57.6%)	1.00
Injured, *n* (%)	111 (23.2%)	61 (25.4%)	50 (21%)	0.28
Close other killed, *n* (%)	225 (47.1%)	117 (48.8%)	108 (45.4%)	0.47
Mean disaster preparedness^c^ (s.d.), range	11.1 (5.6) 0–20	11.3 (5.6) 0–20	11.0 (5.6) 0–19	0.63
Mean depression (ZLDSI^d^) (s.d.), range	12.1 (9.5) 0–39	12.4 (10.0) 0–39	11.9 (9.1) 0–39	0.57
Mean PTSD (MPSS^e^) (s.d.), range	33.9 (31.8) 0–123	35.2 (32.5) 0–120	32.6 (31.2) 0–123	0.36
Mean anxiety (BAI^f^) (s.d.), range	15.6 (13.8) 0–63	16.2 (14.5) 0–60	15.0 (13.1) 0–63	0.31
Mean functional impairment^g^ (s.d.), range	2.5 (1.2) 1–5	2.5 (1.2) 1–5	2.5 (1.2) 1–5	0.93
Mean social cohesion^h^ (s.d.), range	2.7 (1.0) 1–5	2.7 (1.0) 1–5	2.7 (1.0) 1–5	1.00

a Twelve items, adapted from the Humanitarian Emergency Settings Perceived Needs (HESPER) (WHO and King's College, 2011).

b Eleven items, adapted based on the Life Events Checklist (Gray *et al.*, 2004).

c Twenty-item investigator-developed checklist assessing participant report of behaviors conducted to prepare for future disasters.

d Zanmi Lasante Depression Symptom Inventory (ZLDSI) (Rasmussen *et al*., 2015).

e Modified PTSD Symptom Scale (MPSS) (Falsetti *et al*., 1993), translated and adapted for use in Haiti by Kaiser (personal correspondence, June 2014).

f Beck Anxiety Inventory (BAI) translated and adapted by Kaiser *et al*. (2013).

g Five items for women, four items for men, adapted from Kaiser *et al*. (2013).

h Five items, adapted from Sampson *et al*. (1997).

### Data analysis

Please see online Supplementary Appendix 4 for detailed data analysis approach.

## Results

### Participant retention

**Fig. 1.**
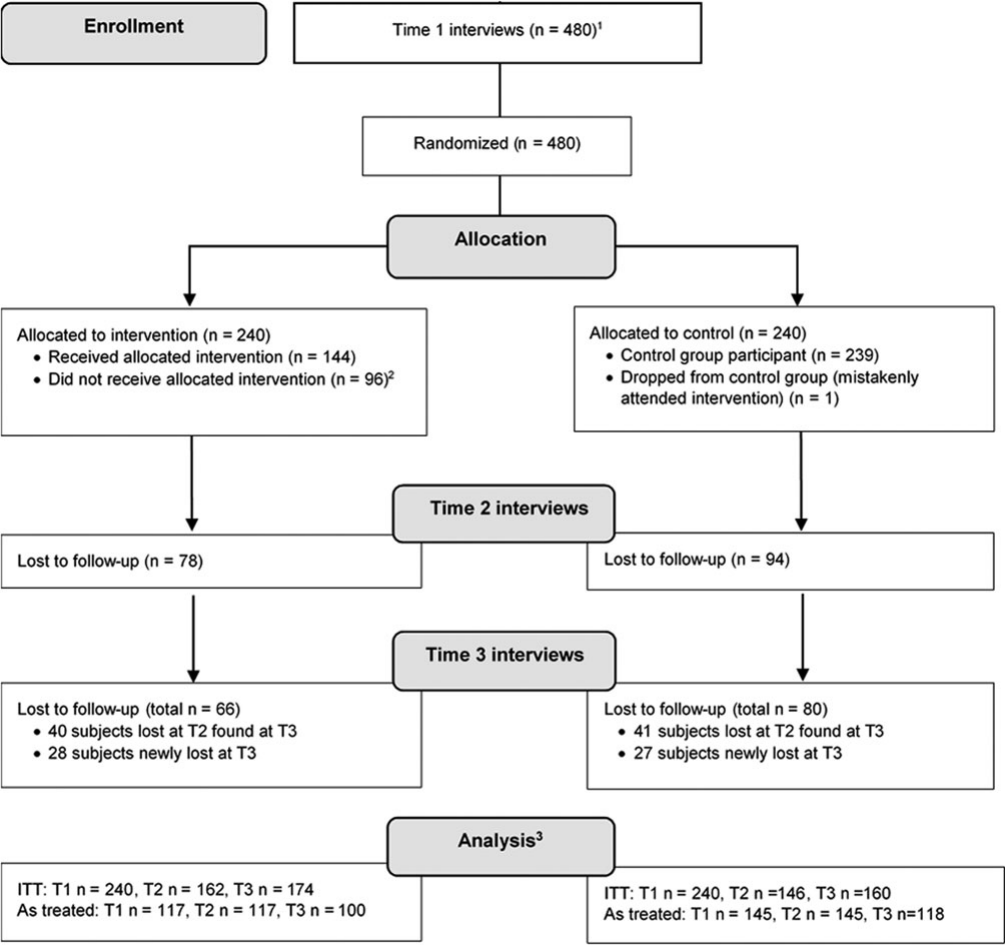
Participant flow diagram. ^1^All willing participants over age 18 were interviewed. An estimated 5% of those approached were not at home (*n* = 24) (note: as there are no accurate public records of dwellings in the area, it is not clear if some of these dwellings were occupied). An estimated 1% of those who were home declined to participate in the study/first interview (*n* = 5). Participants who were not home or declined to participate were replaced with other participants at Time 1. ^2^Some of those who did not attend the intervention were re-contacted for Time 2 interviews (*n* = 23) and gave the following explanations for non-attendance: traveled out of area (26%), had work (22%), childcare responsibilities (13%), unaware of invitation/issues contacting (9%). ^3^Main analyses conducted as intent-to-treat (ITT): all participants analyzed as belonging to the group to which they were initially randomized, regardless of compliance (see Gupta, 2011). In as-treated analyses, only compliant subjects with at least T1 and T2 data were analyzed.

Figure 1 summarizes the flow of participants. Although 480 participants were interviewed at baseline and 240 were randomized to the intervention condition, only 144 participants completed the intervention. At T2, 308 participants were interviewed, and at T3, 334 participants were interviewed.^2^

In light of significant attrition, analyses used an intention-to-treat approach (see Gupta, 2011), adhering to original randomization (i.e. participants randomized to intervention group who did not participate in the intervention were included as intervention participants). Intention-to-treat analyses are generally conservative in terms of estimating treatment effect, and address two major limitations of RCTs: non-compliance and missing outcomes (Gupta, 2011).

### Baseline characteristics

Baseline data, collected at Time 1, for the full sample and for intervention and control groups, are presented in Table 1. Participants were nearly evenly split by gender, and the mean age was approximately 37 years. A quarter of the sample were currently married and on average participants had between two and three children. Almost all were unemployed^3^ and approximately one third identified as students. Nearly all participants were Christian (most commonly Protestant or Catholic). Intervention and control groups were relatively well balanced on demographic variables, although the control group was older (*p* = 0.09) and had more children than the intervention group.

At baseline, the most frequently endorsed chronic stressors were problems with employment, food, and private life. Participants reported high levels of prior disaster exposure, with nearly all exposed to an earthquake and two-thirds exposed to flooding. Most participants (70%) reported significant disaster-related property damage and nearly 60% reported that they had been permanently or temporarily displaced by a prior disaster. Almost half of participants reported that a friend or family member had been killed in a disaster, and nearly a quarter reported that they had been personally injured. There were no significant differences between intervention or control groups in chronic stressors, disaster exposure, or in outcome variables at baseline.

### Intervention effects

See Table 2 for intervention effects. In line with hypotheses 1 and 2, at Time 2 (3–4 months post-intervention), disaster preparedness behaviors significantly increased among intervention participants (moderate to large effect size) while mental health symptoms (depression, anxiety, PTSD full scale and avoidance sub-cluster of symptoms) and functional impairment significantly decreased (small to moderate effect sizes). A trend-level increase in social cohesion was observed among intervention participants (Hypothesis 3). Disaster-related and mental health-related help-giving intention increased following intervention participation (Hypothesis 4). Disaster-related help-seeking increased, but effects on mental health related help-seeking were not significant (Hypothesis 5). Nearly all primary outcomes were still significant at Time 3 (7–8 months post-intervention), with the exception of functional impairment and social cohesion. Disaster-related help-seeking bordered on significance at Time 3.

**Table 2.** Intervention outcomes at Time 2 and Time 3

Variable	Unst intervention effect reg coef, change T1 to T2 (s.e.)	Effect size (Cohen's *d*)	Unst intervention effect reg coef, change T1 to T3 (s.e.)	Effect size (Cohen's *d*)
Disaster preparedness behaviors^a^	4.18*** (0.60)	0.75	2.90*** (0.57)	0.52
Depression	−0.35*** (0.08)	−0.47	−0.21** (0.08)	−0.29
PTSD	−0.46*** (0.10)	−0.49	−0.28** (0.10)	−0.30
Avoidance symptoms of PTSD	−0.35*** (0.10)	−0.41	−0.22* (0.09)	−0.25
Anxiety	−0.27*** (0.07)	−0.41	−0.15* (0.07)	−0.23
Functional impairment	−0.35* (0.13)	−0.29	−0.15 (0.14), NS	−0.12
Social cohesion	0.21 (0.12), *p* = 0.07	0.22	0.03 (0.11), NS	0.03
Help-giving				
Disaster-focused	1.71*** (0.28)	–	1.37*** (0.29)	–
Mental health-focused	2.62*** (0.28)	–	1.39*** (0.25)	–
Help-seeking				
Disaster-focused	0.59* (0.29)	–	0.53 (0.28), *p* = 0.056	–
Mental health-focused	0.20 (0.28), NS	–	0.20 (0.27), NS	–

***p < 0.001, **p < 0.01, *p < 0.05. Unstandardized regression coefficients indicate the change in scale values in the intervention group relative to control, from T1 to T2 or T1 to T3. Cohen's *d* presented for scale variables with three or more items, analyzed as normal Gaussian variables only. All results presented here are intent-to-treat analyses; in all cases, results and corresponding conclusions were qualitatively similar when analyzed as-treated, with the exception of the T1 to T3 change in depression coefficient: −0.16 (0.10), *p* = 0.11, *d* = −0.22; and anxiety coefficient: −0.10 (0.08), NS, *d* = −0.14.

a Because intervention participants were given a radio (see online Supplementary Appendix 1), a separate analysis was conducted wherein an item referencing listening to the radio for disaster warnings was excluded from the disaster preparedness scale; results were qualitatively similar and also statistically significant, indicating intervention effects on outcomes were not dependent upon this compensation.

Results were qualitatively similar for all models when disaster exposure was controlled for by inclusion as a covariate. Gender did not significantly moderate the impact of the intervention for any of our key dependent measures, suggesting that the intervention was equally effective for women and men.

### The role of disaster exposure

In baseline (Time 1) data, there was a trend-level negative correlation between disaster exposure and disaster preparedness (Hypothesis 6). Disaster exposure was positively correlated with mental health measures (depression, PTSD, anxiety, and functional impairment), such that increased exposure was associated with more mental health symptom severity (Hypothesis 7). See Table 3.

**Table 3.** Correlations between variables at baseline

Measure	Disaster exposure	Disaster preparedness	Depression	PTSD	PTSD avoidance	Anxiety	Functional impairment	Social cohesion
Disaster exposure	–	−0.08^a^	0.37***	0.42***	0.37***	0.24***	0.25***	−0.10*
Disaster preparedness	−0.08^a^	–	−0.16**	−0.28***	−0.24***	−0.2***	−0.33***	0.09^a^
Depression	0.37***	−0.16**	–	0.72***	0.46***	0.70***	0.49***	−0.11*
PTSD	0.42***	−0.28***	0.72***	–	0.75***	0.63***	0.44***	−0.11*
PTSD avoidance	0.37***	−0.24***	0.46***	0.75***	–	0.35***	0.34***	−0.16***
Anxiety	0.24***	−0.20***	0.70***	0.63***	0.35***	–	0.46***	−0.07
Functional impairment	0.25***	−0.33***	0.49***	0.44***	0.34***	0.46***	–	−0.06
Social cohesion	−0.10*	0.09^a^	−0.11*	−0.11*	−0.16***	−0.07	−0.06	–

***p < 0.001, **p < 0.01, *p < 0.05.

a Trend-level relationship (*p* < 0.10).

**Fig. 2.**
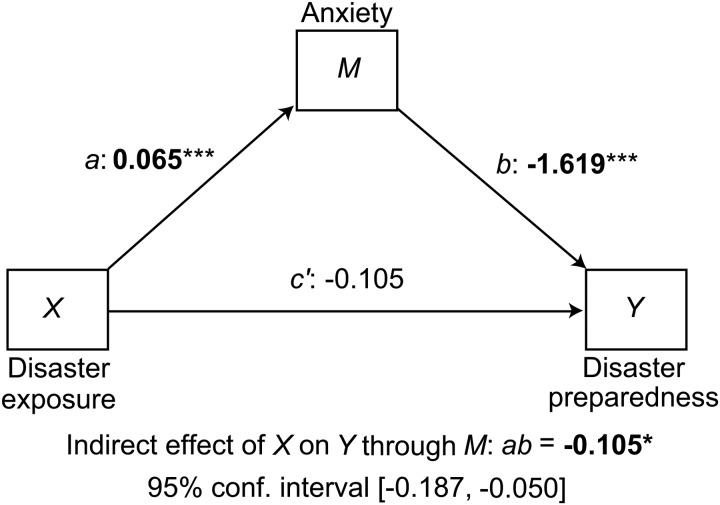
Mediation model diagram: disaster exposure, anxiety, and disaster preparedness. ***p < 0.001, **p < 0.01, *p < 0.05. Note 1. Figure 2 depicts an example of a mediation model (mediating effects of anxiety on the relationship between disaster exposure and preparedness) as visual guide to aid the reader's interpretation of mediation results; data for all other models described in the manuscript can be found in Table 4. In each model, two equations were used: (1) the effect of the independent variable (disaster exposure or the intervention) on the mediator (*a* path), and (2) the effects of the mediator on the outcome variable (*b* path) and the independent variable on the outcome variable (*c’* path). The direct effect of the independent variable on outcomes is given by *c’* and the mediated or indirect effect of the independent variable is given by the product *ab*. The total effect on the outcome is given by *c* (not shown).

Mediation models conducted with Time 1 data revealed that, as predicted, the relationship between disaster exposure and reduced disaster preparedness was mediated by mental health symptoms (*ab*) (Hypothesis 8). Increased disaster exposure was associated with more severe mental health symptoms (*a*), which were in turn associated with poorer disaster preparedness (*b*). Depression, PTSD (overall and avoidance subscale), anxiety, and functional impairment all acted as significant mediators, and in all cases, there was no direct effect of exposure on preparedness (*c’*), indicating full mediation. Social cohesion was not a significant mediator. Disaster preparedness did not mediate a relationship between disaster exposure and mental health symptoms. See Fig. 2 and Table 4.

**Table 4.** Disaster exposure and intervention mediation model results

Mediator	Dependent variable	*a*	*b*	*c'*	*ab*, 95% conf. interval	*ab/c*
*X* = disaster exposure^a^						
Depression	Preparedness	0.117*** (0.015)	−1.123** (0.410)	−0.078 (0.123)	−0.131* (−0.246 to −0.040)	0.626
PTSD	Preparedness	0.171*** (0.019)	−1.767*** (0.319)	0.093 (0.131)	−0.303* (−0.456 to −0.182)	1.446
Anxiety	Preparedness	0.065*** (0.014)	−1.619*** (0.436)	−0.105 (0.125)	−0.105* (−0.187 to −0.050)	0.499
*X* = intervention^b^						
Depression	Preparedness	−0.433*** (0.102)	−1.220* (0.491)	3.444*** (0.773)	0.528*, (0.053 to 1.232)	0.133
PTSD	Preparedness	−0.542*** (0.135)	−1.217** (0.369)	3.314*** (0.762)	0.659*, (0.261 to 1.300)	0.166
Anxiety	Preparedness	−0.349*** (0.095)	−1.981*** (0.520)	3.281*** (0.752)	0.692*, (0.329 to 1.218)	0.174
Preparedness	Depression	3.972*** (0.751)	−0.023* (0.009)	−0.344** (0.107)	−0.090*, (−0.188 to −0.010)	0.207
Preparedness	PTSD	3.972*** (0.751)	−0.039** (0.012)	−0.386** (0.140)	−0.155*, (−0.278 to −0.067)	0.287
Preparedness	Anxiety	3.972*** (0.751)	−0.032*** (0.008)	−0.224* (0.098)	−0.125*, (−0.203 to −0.070)	0.358

***p < 0.001, **p < 0.01, *p < 0.05. Coefficients (and standard errors or confidence intervals) are reported for all paths and for the indirect effect of disaster exposure or the intervention (*ab*), as well as the ratio of indirect to total effects (*ab/c*), a measure of the effect size or proportion of the effect that is mediated.

a The relationship between disaster exposure and preparedness was additionally mediated by the PTSD avoidance subscale [*ab* = −0.220*, (−0.366 to −0.111)], and functional impairment [*ab* = −0.204*, (−0.324 to −0.111)]. Social cohesion was not a significant mediator, and disaster preparedness did not mediate a relationship between disaster exposure and anxiety, depression, PTSD, PTSD avoidance subscale, or social cohesion (data not shown).

b Mediated effects were also present for PTSD avoidance subscale acting as a mediator, *ab* = 0.387*, (0.034–0.992); and as an outcome, *ab* = −0.099*, (−0.217 to −0.012). Neither functional impairment nor social cohesion acted as significant mediators or were mediated by disaster preparedness (data not shown).

### Mechanisms of intervention change

To investigate whether theorized mechanisms of intervention effects were supported by the data, we first explored relationships among variables at baseline. Mental health measures (depression, PTSD, anxiety, and functional impairment) were correlated with disaster preparedness, such that fewer mental health symptoms were associated with more preparedness (Hypothesis 9). Higher social cohesion was associated with better mental health (Hypothesis 10). See Table 3.

We then asked whether the intervention effect on disaster preparedness was mediated by changes in mental health symptoms and/or functional impairment. As expected based upon main intervention results, there was a direct effect of the intervention on preparedness (*c’*). Furthermore, as hypothesized (11), the effect of the intervention on disaster preparedness was partially mediated by changes in depression, PTSD (overall and avoidance subscale) and anxiety (*ab*), such that the intervention decreased mental health symptoms (*a*), and this reduction in symptoms partially explained the increase in preparedness (*b*). There were no mediating effects of functional impairment. In a multiple mediator model involving all three significant mental health mediators acting in parallel, only anxiety remained a significant mediator of the effect of the intervention on preparedness [*ab* = 0.536*, (0.178, 1.086)].

As hypothesized (12), intervention effects on depression, PTSD (overall and avoidance subscale), and anxiety were likewise partially mediated by preparedness, such that the intervention increased preparedness, and heightened preparedness partially explained decreased mental health symptoms. Disaster preparedness did not mediate the intervention's effect on functional impairment. Social cohesion did not mediate effects of the intervention on preparedness or on mental health (Hypothesis 13).

A measure of the size of the mediated effect, calculated as the ratio of indirect effect to total effect (*ab/c*), indicated that mediated effects were greater in general for models in which disaster preparedness mediated the effect of the intervention on mental health, relative to models in which mental health mediated the effect of the intervention on preparedness. Moreover, in both cases, models involving anxiety exhibited the largest mediated effects. See Table 4.

## Discussion

Results indicate that the intervention was effective. Although effect sizes are small to moderate per standard interpretations of Cohen's *d*, considered in light of typical effect sizes in mental health research, and given the challenging context, such results are encouraging. Notably, findings are similar to those in our work with earthquake-affected communities in Nepal, using a similar intervention (Welton-Mitchell *et al*., 2018).

Intervention participation was associated with a significant increase in reported disaster preparedness behaviors (supported by behavioral checks). Intervention participants engaged in an average of four additional preparedness behaviors compared to those in the control group. The intervention also decreased symptoms of depression, PTSD (overall and avoidance subscale), anxiety, and functional impairment, measured using culturally-adapted tools. Intervention participation also resulted in a trend-level increase in perceived social cohesion, and significant increases in help-giving intention, with regard to both mental health and disaster mitigation, and in disaster-related help-seeking. There were no significant effects on mental health related help-seeking, perhaps due to difficulty interpreting this single item; although we had intended to capture even informal help-seeking (family, neighbors), it may have been interpreted as referencing formal mental health services, which are notoriously limited and hard to access in Haiti (WHO, 2010; Khoury *et al*., 2012).

Outcomes (apart from functional impairment and social cohesion) were significant up to seven months after the intervention, suggesting relatively sustainable intervention effects.

In addition to assessing intervention outcomes, data were further analyzed to assess the validity of the underlying theoretical model. These analyses expand upon existing evidence regarding behavioral theories of disaster preparedness, which are primarily derived from developed, western countries (Ejeta *et al*., 2015). First, we investigated the premise that prior disaster exposure can be associated with reduced preparedness, particularly among those with higher mental health symptoms. The correlation matrix, demonstrating a (trend-level) negative relationship between exposure and preparedness, and positive relationships between exposure and mental health symptoms, provided some preliminary support for this idea. Subsequent mediation models provided further support; the negative relationship between disaster exposure and preparedness was explained by increased mental health symptoms (depression, PTSD overall and avoidance subscale, and anxiety) and by reduced functional impairment. These results suggest that chronically disaster exposed individuals may be especially vulnerable to mental health symptoms and related impairment, which may in turn result in low-levels of preparedness (so increasing vulnerability to future disasters). Support for these relationships reinforces the importance of intervention focused on both mental health and preparedness for this population. This finding also contributes a new layer to debate about the role of disaster exposure in predicting preparedness. Whereas prior studies have generally shown positive relationships between prior disaster exposure and preparedness efforts, with some exceptions (for reviews, see Bubeck *et al*., 2012; Kohn *et al*., 2012), these results suggest that, in Haitian populations, mental health symptoms and related impairment may play a key role in determining how exposure influences current preparedness. Future work should explore whether these findings hold in other populations and contexts.

Next, we explored mediated relationships to determine whether the intervention operates in line with hypothesized mechanisms. As expected, intervention effects on preparedness were partly mediated by mental health. Interestingly, symptoms related to three separate manifestations of distress – depression, PTSD, and anxiety – were identified as mediators (as was the PTSD avoidance subscale). This is consistent with research suggesting that symptoms typically associated with depression and other mental health issues may interfere with preparedness (see Eisenman *et al*., 2009). The role of PTSD, and specifically, avoidance symptoms of PTSD, in reduced preparedness, has support in our earlier pilot research in a Haitian IDP camp, in which we found that avoidance symptoms of PTSD, including avoidance of traumatic stimuli associated with prior disasters, are associated with decreased engagement in preparedness (James, 2013). As in other work (Mishra and Suar, 2012; Bodas *et al*., 2017; Wirtz *et al*., 2017), anxiety was also linked to preparedness in this study. Interestingly, of all the mental health variables, anxiety symptoms surfaced as the most reliable factor driving results in associated mediation models (as it carried the largest mediated effect size and remained significant in a multiple mediator model). Further work is needed to illuminate specific symptoms and/or psychological processes (e.g. avoidant coping, self-efficacy) driving results, especially in light of significant overlap among mental health constructs.

Due to lack of prior research on this topic, we also wanted to explore whether data fit statistical models assuming a mediated relationship in the opposite direction. In doing so, we found that intervention effects on mental health were partly mediated by preparedness. These results support a bidirectional model such that the intervention may both influence preparedness through its effects on mental health measures and impact mental health by increasing preparedness. Because we cannot directly manipulate these mediators, we are limited in our ability to conclusively determine direction. However, in the present study, a measure of the size of the mediated effect (*ab/c*) was generally larger for models in which preparedness mediated the effect of the intervention on mental health compared to models in which mental health mediated the intervention's effect on preparedness, suggestive of the potential importance of this putative causal pathway. Moreover, in our work with earthquake-affected communities in Nepal using a similar intervention, mediation models indicated that the effect of intervention on mental health (depression) was partially explained by preparedness, whereas the inverse was not true (Welton-Mitchell *et al*., 2018). Feeling prepared may decrease feelings of anxiety and depression regarding the impact of future disasters, and therefore benefit mental health overall (e.g. Galappatti and Richardson, 2016). Results emphasize the importance of incorporating concrete preparedness training as a strategy for improving well-being among chronically disaster exposed populations.

Results did not support the hypothesized role of functional impairment or social cohesion as mediators of the impact of the intervention on preparedness or on mental health. This may be explained by the relatively weak intervention effects on social cohesion (and to a lesser extent, on functional impairment) in this population.

### Limitations and strengths

Work in highly impoverished, transient, disaster-prone communities invites inevitable challenges. Participant attrition was high, largely due to population transience and initial mistrust of service providers related to a history of low NGO follow-through in the intervention communities. Efforts were made to accommodate for this through use of conservative intent to treat (ITT) analyses, designed to more accurately reflect the impact of an intervention in practice by accounting for treatment non-compliance and dropout in an unbiased manner (Gupta, 2011).

Additionally, logistical and resource challenges meant that local team members served as both facilitators and interviewers. It was therefore not feasible to have interviewers blind to condition, creating the potential for bias (although importantly, team members did not interview the same participants who were in their intervention groups). Moreover, this study entailed comparison to a wait-list control group. Future studies should compare this mental health-integrated disaster preparedness model to disaster preparedness as usual and mental health as usual models, to determine whether the combination of elements is in fact more effective than each element administered alone.

Future work should also test adapted versions of this intervention in other cultural and disaster contexts. It is possible that integration of mental health and disaster preparedness interventions may be more critical in certain contexts, such as those in which disasters are chronic, mental health problems are prevalent and communities may feel especially hopeless, avoidant, or anxious about preparedness. Of note, two related studies were conducted in Nepal, using a parallel intervention adapted to flood- and earthquake-exposed communities. Whereas preparedness and social cohesion results are generally consistent, mental health results do appear to vary across contexts, with more robust results in earthquake-affected communities (Welton-Mitchell *et al*., 2018).

Despite limitations, this study demonstrated important results. A multinational team developed an innovative, theory-based intervention incorporating mental health in a disaster preparedness framework. This brief, low-cost model was facilitated by local lay mental health workers, and as such, is likely to be feasible in a variety of low-resource humanitarian contexts. The model was tested using a rigorous RCT design, culturally-adapted and validated measures, and analyzed using a thorough and conservative approach.

## Conclusion

Reviews examining the state of the evidence regarding mental health and psychosocial intervention in disaster and other humanitarian contexts have emphasized that research is needed regarding the effectiveness of low-cost, low-intensity (brief, non-invasive) psychosocial interventions; group-based interventions; and interventions using natural support systems, evaluated using scientifically rigorous methods (Blanchet *et al*., 2013). The current study directly addresses these gaps, building the evidence base through evaluation of a theory-based intervention implemented in the aftermath of disaster, designed to enhance community preparedness for future disasters. Research revealed effects on disaster preparedness, mental health symptoms, and helping behaviors, most of which were maintained more than 7 months post-intervention. Mediation models also helped to illuminate likely underlying mechanisms of change, consistent with hypotheses linked to theoretical models. This mental health integrated disaster preparedness intervention has the potential to be scaled up for use in numerous contexts, with the aim of mitigating disaster impact among especially vulnerable populations by prioritizing prevention and expediting recovery.

## Footnotes

† The notes appear after the main text.

## Notes

1 We focus on manifestations of distress most common in populations exposed to disaster (Norris *et al.* 2002; Foa *et al*. 2006; Cerdá *et al*. 2013) and use culturally-adapted symptom scales as proxies for level of distress, rather than as diagnostic tools. The avoidance subscale of PTSD is included as a separate outcome variable due to hypothesized role of avoidance symptoms in decreasing preparedness.

2 Per local researchers, reduced intervention and interview participation was largely explained by difficulty re-contacting invitees due to changing phone numbers and transient residence. Some participants who were unreachable at Time 2 were contacted at Time 3. See Figure 1.

3 As recruitment was conducted during the day, it is possible that individuals who are unemployed (and therefore at home) are over-represented in this sample. However, unemployment rates described here are consistent with those shared by community leaders.

## Supplementary material

For supplementary material accompanying this paper visit https://doi.org/10.1017/S0033291719000163. click here to view supplementary material

## Availability of data and material

The datasets generated and/or analyzed during the current study are being prepared for the following repository: data.gov.uk (Find open data service). In the meantime, those interested can contact the authors for access to the datasets.

## Trial registration

Registered with Clinical Trials Registry-India, National Institute of Medical Statistics. Registration number: CTRI/2018/02/012002. Date of registration: 02/20/2018 (retrospective): http://ctri.nic.in/Clinicaltrials/advsearch.php.
