# Supplementary material for: Integrating mental health and disaster preparedness in intervention: a randomized controlled trial with earthquake and flood-affected communities in Haiti
Source: Psychol Med. 2019 Feb 14;50(2):342–52. doi: 10.1017/S0033291719000163 (PMC7083573; doi:10.1017/S0033291719000163)
Supplement: Supplementary file 1 [file S0033291719000163sup001.docx]

**Online Appendices**

**Appendix 1:** *Participants and Procedures*

Port-au-Prince was chosen as the research site due to its extreme vulnerability to disasters. In addition to the massive 2010 earthquake that killed an estimated 150,000 and left almost 2.3 million people homeless (Kolbe et al. 2010; Desmarais/IOM, 2011; Daniell et al. 2013), Haitians have weathered multiple deadly hurricanes and frequent flooding and landslides. Nearly 40,000 families were affected by Hurricane Sandy in 2012 (USAID, 2013) and 2.2 million people were affected by Hurricane Matthew in 2016 (World Bank, 2017). Haiti’s environmental vulnerability is compounded by poverty, associated deforestation and lack of infrastructure, and by a long history of political instability, violence, and corruption (Transparency International, 2017).

Chronic disaster exposure has increased Haitians’ vulnerability to future disasters. In Port-au-Prince, years after the 2010 earthquake, many Haitians still live in unstable structures on hills and flood-plains, some in areas that originated as IDP camps in the initial aftermath of the earthquake. Seasonal hurricanes and associated heavy rains, flooding, and landslides impact residents on a yearly basis (IOM, 2017).

Research included 480 community members, drawn from three affected communities (160 participants in each) in metropolitan Port-au-Prince between July 2014 and April 2015. Identification of research communities was informed by a review of locations within metropolitan Port-au-Prince experiencing natural disasters, specifically flooding and landslides over the past 5 years. Relevant stakeholders were also consulted, including local community-based partners (Soulaje Lespri Moun) and community leaders in each location. Ultimately, three communities were chosen, located within Cite Soleil, Carrefour, and Bon Repos. All are in flood prone areas with many community members living in make-shift dwellings, formed from low-quality cement and flimsy or scavenged materials including low quality wood and plastic sheeting. Two of the three areas consist of semi-permanent structures initially constructed in the aftermath of the 2010 earthquake.

In the selected communities, the research team collaborated with local community leaders to develop the sampling frame from which households were invited to participate in the research by trained Haitian interviewers. To recruit a representative sample covering a sizeable area, interviewers approached every fifth dwelling in the order encountered when walking through the community, starting from the side of the community most affected by flooding in past seasons. At each household, researchers used a recruitment script to assess interest and eligibility (age 18-65; household decision-maker; availability to attend 3-day intervention training). There were no specific screen out or in criteria, assuming the community member was able to give consent. All eligible community members were invited to participate (one adult from each household). Attempts were made to ensure equal gender representation by requesting to speak with either a woman household decision-maker or a man household decision-maker at every other dwelling. In most households approached, an eligible community member agreed to participate in the interview. In a few cases, household members declined to participate, due to work or childcare responsibilities.

During baseline interviews, all potential participants, including those who declined to participate, received drinks and snacks as compensation. This is consistent with compensation offered in similar settings in Haiti. Compensation was kept minimal at baseline to reduce the likelihood of coercive influence. Intervention participants received meals, materials (printed documents, note-taking materials), and a small gift likely to be useful during future natural hazards (a radio). At Time 2 and 3 interviews, all participants received snacks, drinks, and small gifts related to disaster relief (e.g., shovels, rakes, rubber boots)^[[1]](#footnote-1)^.

**Appendix 2:** *Intervention description*

Mental health integrated disaster preparedness intervention content was developed in collaboration with Haitian team members, including discussion about cultural and religious attributions for disasters and mental health reactions, mental health related stigma in Haiti, and activities and coping strategies derived from popular Haitian games and pastimes (see James et al. 2018; Welton-Mitchell & James, 2018 for details). This approach to intervention development is consistent with best practice guidelines and recommendations from others working in Haiti (see Ferrer-Wreder et al. 2012; Guerda et al. 2015).

The content of the sessions is based on a standardized manual (James et al. 2016, publicly available online in English and Haitian Kreyol). Day 1 includes discussion about mental health and psychosocial reactions to disaster-related stress, and teaching associated coping strategies, including skills to reduce potential avoidance of disaster-related material (e.g., self-calming through breathing, grounding, mindfulness, and muscle relaxation exercises). On Day 2, the workshop transitions to focus on disaster preparedness, including facilitated discussions regarding links between common attributions for disasters (natural causes, God’s will) and preparedness motivation. Facilitators introduce common scientific explanations for disasters such as earthquakes and floods and share recommended preparedness strategies. This is done without discouraging pre-existing cultural and religious beliefs which participants are encouraged to maintain alongside new information. At the end of Day 2 and moving into Day 3, participants practice providing disaster and mental health related peer support to one another, including through a “mini disaster simulation” in which participants demonstrate skills learned throughout the 3 days. Additional detail is available in other publications (James et al. 2018; Welton-Mitchell & James, 2018; Welton-Mitchell et al. 2018). Lay mental health workers were trained by PIs (both mental health professionals) in an intensive week-long session consisting of manual review and role-play.

**Appendix 3:** *Measures*

Haitian researchers employed by Soulaje Lespri Moun conducted participant interviews in Haitian Kreyol. Unless otherwise noted, measures described below were translated to Haitian Kreyol by members of the research team, with back-translation and piloting to ensure items were adequately understood and that constructs were equivalent across languages.

**Demographics** included age, marital status, number of children, employment, education, and religion.

Exposure to **chronic stressors** was assessed at baseline using 12 items adapted from the Humanitarian Emergency Settings Perceived Needs (HESPER) (WHO & King’s College, 2011), the impact of which were assessed using a three-point scale (Cronbach’s α = 0.84).

**Disaster exposure** was assessed at baseline through 11 items (e.g*., experienced an earthquake; experienced flooding; displaced from home due to a disaster*), each with yes/no response options, adapted based on the Life Events Checklist (Gray et al. 2004). Items were summed to create a measure of overall exposure (Cronbach’s α = 0.64).

Self-reported **disaster preparedness** was measured at all time points using a 20-item investigator-developed checklist in which participants were asked to indicate which behaviors they had done to prepare for future disasters (e.g., *Made a disaster supply kit; put important documents in a safe place; secured dwelling/made stronger in some way; discussed a family evacuation plan or where to meet up if separated; considered safe and vulnerable places in your community*). Items in checklists were developed through focus groups and interviews conducted as part of the authors’ prior disaster research in metropolitan Port-au-Prince (James, 2013). Researchers minimized the potential for bias associated with self-report by asking to see evidence of preparedness during household interviews. Sums of selected items were calculated for each participant. Reliability of this formative measure was acceptable (test-retest reliability from Time 1 to Time 2 in control subjects only, Pearson *r* = 0.50, *p* = 5.4 x 10-8).

**Depression symptoms** were assessed using the 13-item Zanmi Lasante Depression Symptom Inventory (ZLDSI) in Haitian Kreyol, developed by Rasmussen and colleagues (2015). The ZLDSI was designed to measure culturally-bound idioms of distress among Haitians, for use by Zanmi Lasante/Partners in Health as a depression screener in their Haitian mental health clinics. The instrument has demonstrated high levels of internal reliability, construct reliability both regarding compatibility with local symptom categories and measures of depression in other global mental health research, and concurrent validity in terms of functional impairment (Rasmussen et al. 2015). Items used a four-point response scale assessing frequency of depression reactions during the past 15 days (1 = Never; 4 = Almost every day/10-15 days) (Cronbach’s α = 0.93).

**Posttraumatic Stress symptoms** were assessed using the 17-item Modified PTSD Symptom Scale (MPSS) (Falsetti et al. 1993), also translated to Kreyol and adapted for use in Haiti by Kaiser (personal correspondence, June 2014). The MPSS consists of 17-items assessing symptoms associated with a past traumatic event as experienced over the past two weeks. Each symptom consists of a 4-point assessment of frequency (0 = Not at all; 3 = Almost always), followed by a 5-point assessment of severity (0 = Not at all stressed; 4 = Very very stressed) (Cronbach’s α = 0.96). Due to the hypothesized role of avoidance symptoms associated with PTSD in intervention response, an **avoidance subscale** was also developed for testing, consisting of the mean of items measuring frequency and severity of 1) efforts not think about traumatic events and 2) efforts to avoid situations and activities reminding one of traumatic events.

**Anxiety** symptoms were assessed using the 21-item Beck Anxiety Inventory (BAI) translated to Kreyol and adapted to the Haitian context (Kaiser et al. 2013), with demonstration of good internal and construct validity in Haiti. Items assessed frequency that participants were bothered by symptoms in the past two weeks using a four-point response scale (0 = Not at all; 3 = I couldn’t bear it) (Cronbach’s α = 0.94).

**Functional impairment** was assessed using a selection of items from a scale developed for use in Haiti (Kaiser et al. 2013) to assess difficulties during the prior two-weeks among men and women. Five items were used for women (e.g., problems with cleaning the house, taking care of your family) and 4 items were used for men (e.g., problems with jobs, supporting your family). Items used a 5-point response scale (male Cronbach’s α = 0.78; female Cronbach’s α = 0.83).

**Social cohesion** was assessed using five items (e.g. *People in this community are willing to help their neighbors*) on a 5-point scale (adapted from Sampson et al. 1997) (Cronbach’s α = 0.76).

**Help-giving** was assessed using two 2-item investigator-developed scales, one focused on mental health (*If you encountered someone with mental health problems in your community would you help them in some way?* and *Do you feel you have the skills to help someone with mental health problems?*) and the other on disaster preparedness/response (*Do you think you would be willing to help others in your community to 1) prepare for or 2) respond to a disaster?*). All items used a Yes/No response format. Each pair of items was summed.

**Help-seeking** was assessed using one investigator developed item focused on mental health (*Would you be comfortable seeking help from others if you were struck by sadness or mental tension that made your life difficult?,* drawing on work by Kohrt & Hruschka, 2010) and one focused on disasters (*Would you be comfortable seeking help from others if you needed something to prepare for or in the aftermath of a disaster?*) on a 4-point scale.

**Appendix 4:** *Data Analysis*

We assessed the comparability of the control and intervention groups with t-tests, and Fisher’s exact test for categorical demographic data. These, and correlation analyses of scales, utilized all Time 1 data.

For main intervention effect analyses, an intent-to-treat approach was taken, wherein subjects were analyzed as belonging to the experimental condition they were initially randomized to regardless of their actual participation. These were followed by as-treated analyses, wherein only treatment compliant subjects for whom data was available at both Times 1 and 2 were included. Depression, PTSD, anxiety, functioning, and social cohesion scale responses were averaged, with responses on other scales summed. Subjects’ data for averaged scales were included in analyses if at least two-thirds of scale items were available.

Data were analyzed in a three-level linear mixed effects model (measurements across time clustered within participants clustered within community), with fixed effects interactions of time point and intervention and random intercepts at community and participant level. In some cases, no third-level clustering at the community level was included, when it did not improve model fit.

Scale measures were approximately Gaussian distributed and no (i.e., identity) link function was employed; summed binary yes/no help-giving questions (help-giving/seeking items) were analyzed using a binomial logit-link model, and single-question ordinal help-seeking questions were analyzed by cumulative logit-link. Treatment effect coefficients represent the change between time points in the intervention group relative to the control group. Cohen’s D values were calculated by dividing coefficients by standard deviation of dependent measures at Time 1 (Gaussian-distributed variables only).

Mediation models were conducted to explore the relationship between disaster exposure and disaster preparedness and mental health, as well as the mechanisms by which the intervention acted. Bias-corrected confidence intervals for indirect effects were calculated by bootstrapping of linear regression coefficients as recommended by Hayes (2012) using 50,000 resamples. Time 1 data were used for disaster exposure mediation models; change scores for respective scales from Time 1 to Time 2 were used for intervention effect analyses (only subjects with data at both timepoints included). In the case that multiple mediators were identified for a particular dependent measure for intervention effect mediation models, they were entered into a simultaneous multiple parallel mediator model to assess the robustness of their relative contribution. A measure of effect size or proportion of the total effect that acted through the mediating variable was calculated as the ratio of indirect to total effect sizes, or *ab / c*.

References

**Daniell JE, Khazai B, Wenzel F** (2013) Uncovering the 2010 Haiti earthquake death toll. *Natural Hazards and Earth System Sciences Discussions* **1**, 1913-1942.

**Desmarais D/IOM** (2011) Report of the United Nations in Haiti 2010: Situation, Challenges and Outlook. ([http://www.un.org/en/peacekeeping/missions/minustah/documents/un_report_haiti_2010_en. pdf](http://www.un.org/en/peacekeeping/missions/minustah/documents/un_report_haiti_2010_en.%20pdf)). Accessed January 2018.

**Falsetti SA, Resnick HS, Resick, PA, Kilpatrick DG** (1993) The modified PTSD symptom scale: a brief self-report measure of posttraumatic stress disorder. *The Behavior Therapist* **16,** 161-162.

**Ferrer-Wreder L, Sundell K, Mansoory S** (2012) Tinkering with Perfection: Theory Development in the Intervention Cultural Adaptation Field. *Child & Youth Care Forum*, **41**, 149-171.

**Gray MJ, Litz BT, Hsu JL, Lombardo TW** (2004) Psychometric properties of the life events checklist. *Assessment* **11**, 330-341.

**Guerda N, Kahaema B, Ho K** (2015) Implementing culturally sensitive and sustainable mental health training programs internationally: lessons from Haiti. *International Journal of Culture and Mental Health* **8**, 446-457.

**Hayes AF** (2012) PROCESS: A versatile computational tool for observed variable mediation, moderation, and conditional process modeling [White paper]. (<http://www.afhayes.com/public/process2012.pdf>). Accessed January 2018.

**International Organization for Migration** (2017, January) Humanitarian aid: 7 years after the 2010 earthquake, who remains displaced? (<http://haiti.iom.int/humanitarian-aid-7-years-after-2010-earthquake-who-remains-displaced>). Accessed January 2018.

**James LE** (2013) A Pilot Assessment of Psychological Factors Associated with Hurricane Sandy Preparedness in Post-earthquake Haiti. *Quick Response Grant Report series*, *Natural Hazards Center, Institute of Behavioral Sciences, University of Colorado, Boulder* **246**, 1-15.

**James LE, Welton-Mitchell C, Khanal SN** (2018). Mental health integrated disaster preparedness (MHIDP) in Nepal and Haiti. Mental Health Innovation Network (<http://www.mhinnovation.net/innovations/mental-health-integrated-disaster-preparedness-mhidp-nepal-and-haiti?qt-content_innovation=0#qt-content_innovation>).

**James LE, Welton-Mitchell C, Soulaje Lespri Moun** (2016). Community-based disaster mental health intervention (CBDMI): Curriculum manual for use with communities affected by natural disasters in Haiti (<http://www.elrha.org/researchdatabase/community-based-disaster-mental-health-intervention-cbdmhi-curriculum-manual-use-communities-affected-natural-disasters-haiti/>).

**Kaiser B, Kohrt BA, Keys H, Khoury NM, Brewster A** (2013) Strategies for assessing mental health in Haiti: Local instrument development and transcultural translation. *Transcultural Psychiatry* **50**, 532 – 558.

**Kohrt BA, Hruschka DJ** (2010) Nepali Concepts of Psychological Trauma: The Role of Idioms of Distress, Ethnopsychology, and Ethnophysiology in Alleviating Suffering and Preventing Stigma. *Culture, Medicine and Psychiatry* **34,** 322-352.

**Kolbe AR, Hutson RA, Shannon H, Trzcinski E, Miles B, Levitz N, Puccio M, James L, Noel JR, Muggah R** (2010) Mortality, crime and access to basic needs before and after the Haiti earthquake: a random survey of Port-au-Prince households. *Medicine, Conflict and Survival* **26**, 281-297.

**Rasmussen A, Eustache E, Raviola G, Kaiser B, Grelotti DJ, Belkin GS** (2015) Development and validation of a Haitian Creole screening instrument for depression. *Transcultural Psychiatry* **52**, 33-57.

**Sampson RJ, Raudenbush SW, Earls F** (1997) Neighborhoods and violent crime: A multilevel study of collective efficacy. *Science* **277**, 918-924.

**Transparency International** (2017) Corruptions Perception Index 2017. (https://www.transparency.org/country/HTI). Accessed April 2018.

**United States Agency for International Development (USAID)** (2013) *Haiti – Hurricane Sandy. Fact Sheet #1 Fiscal Year 2013, February 15, 2013.* (https://www.usaid.gov/documents/1866/haiti-hurricane-sandy-fact-sheet-021513). Accessed January 2018.

**Welton-Mitchell C, James L** (2018). Evidence-based mental health integrated disaster preparedness in Nepal and Haiti. *Special Edition of the Humanitarian Exchange on Mental Health and Psychosocial Support in Humanitarian Response.* Guest editors: Wendy Fenton and Anne Harmer.

**Welton-Mitchell, C, James, L, Khanal, SN, James, A** (2018). An integrated approach to mental health and disaster preparedness: a cluster comparison with earthquake affected communities in Nepal. *BMC Psychiatry* **18,** 296

**World Health Organization (WHO), King’s College** (2011) *The Humanitarian Emergency Settings Perceived Needs Scale (HESPER): Manual with Scale*. Geneva: World Health Organization.

**World Bank** (2017). Rapidly assessing the impact of Hurricane Matthew in Haiti. *Result Briefs.* (<http://www.worldbank.org/en/results/2017/10/20/rapidly-assessing-the-impact-of-hurricane-matthew-in-haiti>). Accessed January 2018.

1. Compensation is higher at these time points than at baseline to reward participants for cumulative time spent on project tasks and because coercion is less of a concern once participants have already committed to participation. [↑](#footnote-ref-1)
